# Functional diversity of woody plant communities and its driving factors in the northern Hainan volcanic lava region

**DOI:** 10.3389/fpls.2026.1888449

**Published:** 2026-07-29

**Authors:** Siyi Li, Shuhai Zhuo, Bijia Zhang, Xuena Wan, Han Wang, Wei Lin, Hong Yang, Peng Zhou, Jinrui Lei

**Affiliations:** 1 School of Tropical Agriculture and Forestry (School of Agriculture and Rural Affairs, School of Rural Revitalization), Hainan University, Haikou, China; 2 Hainan Academy of Forestry (Hainan Academy of Mangrove), Haikou, China; 3 Key Laboratory of Tropical Forestry Resources Monitoring and Application of Hainan Province, Haikou, China

**Keywords:** environmental drivers, functional diversity, leaf functional traits, northern Hainan region, volcanic lava region

## Abstract

**Objective:**

To assess woody plant community functional diversity and its main environmental drivers in the northern Hainan volcanic lava region, a tropical volcanic landscape with marked habitat heterogeneity.

**Methods:**

Woody plant communities were surveyed in the field, leaf functional traits were measured, and nonparametric tests, correlation analysis, redundancy analysis (RDA), and structural equation modeling (SEM) were used to evaluate functional-diversity patterns and potential direct and indirect driver pathways.

**Results:**

Community-weighted mean values of leaf area (LA), leaf dry matter content (LDMC), chlorophyll a/b ratio (Chl a/Chl b), leaf dry weight (LDW), and leaf tissue density (LTD) were higher in trees than in shrubs (*p* < 0.001). Functional richness (FRic), functional dispersion (FDis), and Rao’s quadratic entropy (RaoQ) were also higher in trees (*p* < 0.001). Mean annual precipitation (MAP), soil pH, and aggregation index (AI) were the main drivers for the whole community, tree layer, and shrub layer, respectively. SEM suggested that meteorological and environmental factors, anthropogenic-disturbance, and landscape factors may affect functional diversity both directly and indirectly through soil properties.

**Conclusion:**

Functional diversity in this volcanic lava ecosystem is shaped by multiple environmental pathways, with soil properties playing an important mediating role. These findings provide evidence for conserving and managing woody plant communities in tropical volcanic landscapes.

## 1 Introduction

With continued progress in community and functional ecology, trait-based functional diversity has become an important link between biodiversity and ecosystem functioning (Petchey and Gaston, 2006). Compared with traditional species-richness metrics, functional diversity better connects organisms, communities, and ecosystems by capturing the composition and variation of species functional traits across ecosystems (Petchey and Gaston, 2006), and thereby provides a more comprehensive description of ecosystem functioning and strongly influencing the stability of populations, communities, and ecosystems (Sheng, 2015). Therefore, understanding plant functional diversity is essential for clarifying the complex relationships between biodiversity and ecosystem functioning (Zhao et al., 2022).

Patterns of functional diversity are not random; they are shaped jointly by multiple ecological processes and environmental drivers. Consequently, identifying the mechanisms underlying community formation has become one of the central aims in ecological research (Lhotsky et al., 2016). Plant functional diversity refers to the value range and spatial distribution of functional traits that influence ecosystem functioning (Hao et al., 2022). Earlier research mainly relied on taxonomic or species-level approaches, which limited the ability to identify the core mechanisms that drive community assembly (Götzenberger et al., 2012). In recent years, studies of plant functional traits have become a major focus in ecology, and analyses of community-level trait distributions allow researchers to quantify the strength of the constraints that govern community assembly under different environmental drivers (Swenson et al., 2011). At regional scales, community structure is mainly determined by two deterministic processes: environmental filtering and limiting similarity (Zhu et al., 2020). Environmental filtering is a deterministic process in which abiotic factors select taxa with particular trait profiles, allowing them to establish and persist, thereby increasing functional trait similarity among coexisting taxa, which is expressed as phenotypic or trait convergence (Lavorel and Garnier, 2002). Limiting similarity suggests that species with strong niche overlap are unlikely to coexist, and that limits on ecological similarity promote greater functional differentiation among species, resulting in trait divergence (Zuo et al., 2021). Previous studies have shown that these two processes operate together under different environmental conditions, and both processes have been widely observed in Chinese forest communities (Wang et al., 2012). However, because of the complexity of multi-scale environmental drivers and underlying ecological processes, a clear consensus on the trait-mediated mechanisms governing community assembly is still lacking (Götzenberger et al., 2012). Therefore, investigating interactions among functional traits, environmental drivers, and functional diversity provides a critical lens for clarifying the mechanisms underlying biodiversity maintenance and ecosystem complexity.

Multiple environmental drivers interact to shape plant community diversity patterns, providing insights into how environmental gradients structure biological communities (Chapman and McEwan, 2018). Extensive evidence shows that microtopographic and soil factors act as major drivers of community assembly at fine spatial scales, whereas macroclimatic factors and regional elevational gradients have stronger effects at broader spatial scales (Zu et al., 2019; Liang et al., 2023). Although plant growth and fitness are fundamentally shaped by local environmental conditions, specific environmental drivers—such as macroclimatic regimes, soil properties, anthropogenic disturbances, and spatial landscape configurations—have emerged as central topics in contemporary research on plant functional diversity. Macroclimatic factors strongly influence community-level functional diversity and the underlying trait structure (Valencia et al., 2015). By altering the fitness of dominant and rare phenotypes, climatic factors may alter the distribution of community functional traits and thereby modulate functional diversity (Xu et al., 2024). Accumulating evidence shows that precipitation regimes govern plant water-use strategies and ecological niche partitioning, thereby shaping community-level functional diversity (Zuo et al., 2021; Wang et al., 2022). Environmental drivers, including topography and soil properties, drive shifts in the spatial arrangement of plant functional traits and ultimately shape community functional diversity (Ding et al., 2011). Anthropogenic disturbance is considered a key factor affecting the functional structure of plant communities, because it may affect functional diversity by altering resource availability, habitat conditions, and community composition (Mouillot et al., 2013). Moreover, increased landscape fragmentation, higher patch density, and reduced landscape connectivity may intensify dispersal limitation and habitat filtering, thereby reducing the functional diversity of sandy-land plant communities, whereas a higher proportion of large habitat patches may help maintain regional functional trait diversity (Cao et al., 2023). Across broad to local scales, climatic conditions, anthropogenic disturbances, topography, and soil variables collectively shape functional trait patterns (Liu and Ma, 2015). In summary, plant community functional diversity shows complex spatial patterns across environmental gradients, jointly regulated by the dual forces of environmental filtering and limiting similarity, which interact with the direct and indirect pathways of multi-scale environmental drivers. Comprehensive analyses integrating multidimensional traits, multi-species interactions, and diverse community types are important for clarifying the deterministic processes of community assembly, thereby improving our understanding of ecological functioning and providing a theoretical basis for the adaptive conservation and management of plant communities (Liu et al., 2020).

Currently, studies on plant functional diversity have mainly focused on large-scale global patterns, small-scale local patterns, and community-level processes (Yao et al., 2021), whereas studies of functional diversity in volcanic lava regions remain limited. The Leiqiong region, comprising the Leizhou Peninsula and Hainan Island, is one of the regions in China that has experienced the most intense and persistent volcanic activity since the Cenozoic (Duan et al., 2021). Following volcanic eruptions, lava flows destroy the original vegetation and alter soil conditions, initiating primary succession on bare rock surfaces. Consequently, volcanic lava regions exhibit a shorter successional history than the surrounding landscapes (Shen et al., 2019). Currently, studies on plant diversity in the volcanic lava region of northern Hainan have mainly focused on the discovery of new species (Zhang et al., 2015), species diversity and phylogenetic diversity among vascular plants (Yuan et al., 2017), and the structural properties of plant-pollinator networks (Zeng et al., 2022), whereas research on the distribution patterns and drivers of plant community functional diversity in this region remains insufficient. Based on this theoretical framework and the characteristics of the study region, this study was designed to address the following key questions: (1) How does functional diversity differ between the tree and shrub layers in the volcanic lava region? (2) How is functional diversity related to drivers such as soil properties, meteorological and environmental factors, anthropogenic disturbance, and landscape factors? (3) Through which pathways do the key drivers affect functional diversity? Using leaf functional-trait data collected from plant communities across the volcanic lava region of northern Hainan, this study examined the spatial distribution in functional traits in woody plant communities from the perspective of leaf functional traits and functional diversity, assessed the relationships between functional diversity and its driving factors, and explored the potential pathways of key drivers on plant functional diversity, aiming to provide an empirical basis for elucidating plant community assembly mechanisms, ecosystem management, and sustainable resource use in volcanic lava regions.

## 2 Materials and methods

### 2.1 Study area description

**Figure 1:**
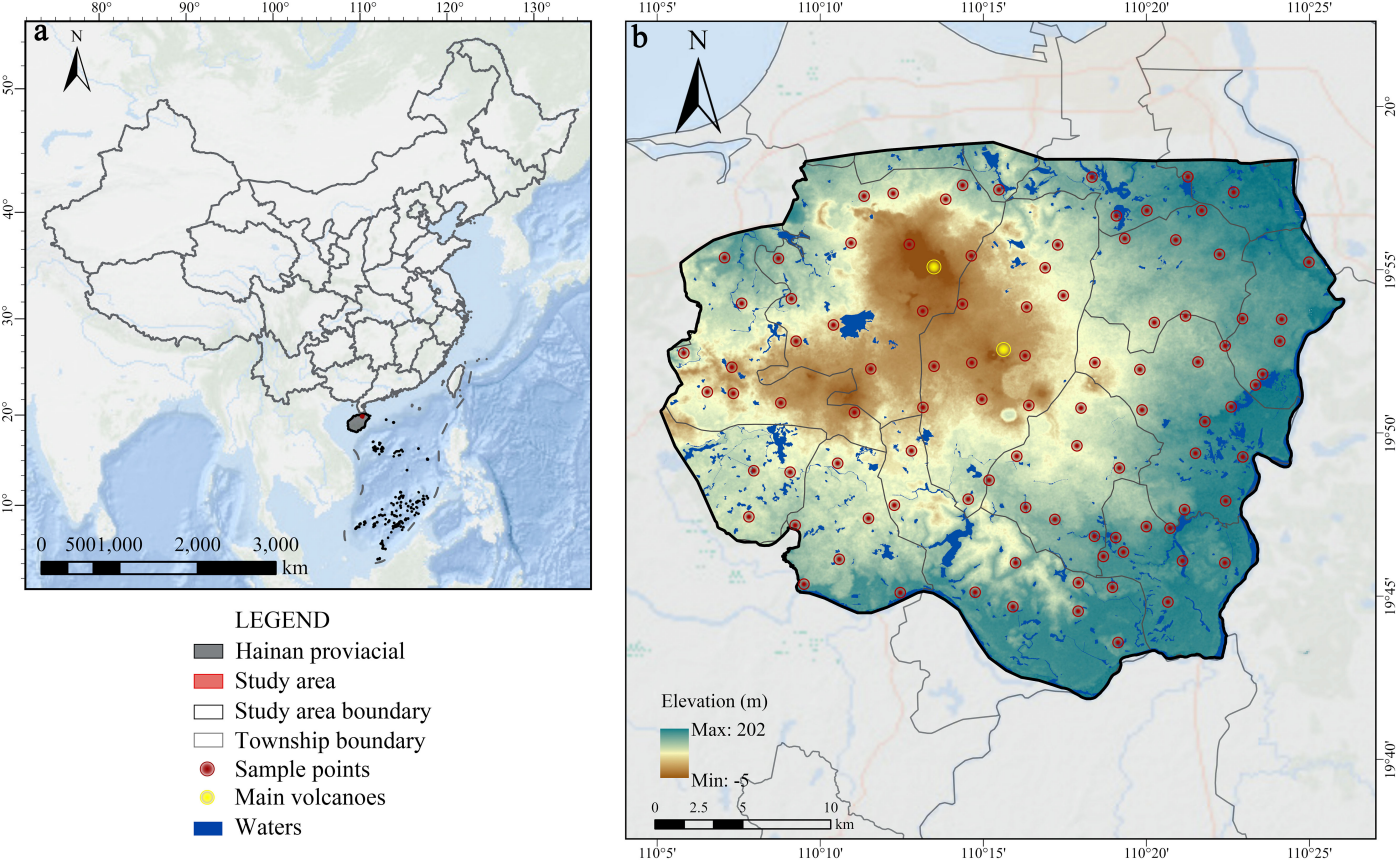
Locations of the study area and distribution of sampling points.

The study was conducted in a representative volcanic lava area located in northern Hainan Island, China (19°42′–20°00′N, 110°05′–110°26′E), extending east to the banks of the Nandu River, west to Laocheng Town in Chengmai County, north to the urban area of Haikou City, and south to the Nandu River Basin, covering a total area of approximately 817.97 km^2^ (**Figure 1**). This area has a representative monsoon climate typical of the northern edge of the tropics, featuring clearly separated wet and dry seasons. The dry season extends from November to April and is characterized by marked water deficits and low precipitation, whereas the rainy season lasts from May to October and is characterized by concentrated and abundant rainfall. The study area has a mean annual temperature of 23.8 °C, a mean annual precipitation of 1,639 mm, and a mean relative humidity of approximately 85%. The study area is mainly characterized by volcanic platforms consisting of Cenozoic basalt and pyroclastic rocks, forming part of the northern Hainan volcanic cluster. The soils are mainly laterite derived from the weathering of volcanic rocks, which is characterized by a loose texture, high porosity, and high permeability. Moreover, the soil is spatially heterogeneous. The volcanic lava region of northern Hainan contains abundant plant resources and harbors diverse valuable germplasm resources. The native vegetation in the study area is well developed and dominated by tropical seasonal rainforests. The presence of dominant woody species, including *Litchi chinensis*, *Dimocarpus longan*, *Atalantia buxifolia*, and *Psychotria asiatica*—forms a distinct tropical vegetation community adapted to volcanic habitats.

### 2.2 Sample plot setting and survey

In areas with typical plant communities within the volcanic lava region of northern Hainan, 96 tree plots (10 m × 10 m) were established, and all individuals in the tree layer (diameter at breast height ≥ 2 cm) were surveyed and measured, with species identity, abundance, diameter at breast height, canopy coverage, and height recorded for each tree. In each tree plot, four shrub subplots (5 m × 5 m) were established at the four corners to record shrub species composition, height, abundance, and coverage, while also recording site-level information such as elevation and geographic coordinates. Species were identified, classified, compiled, and analyzed according to the Angiosperm Phylogeny Group (APG) classification system and relevant botanical records in Flora Reipublicae Popularis Sinicae (Editorial Committee of FRPS, 1959–2004).

### 2.3 Soil sample collection and analysis

Soil samples were collected using a five-point method, with pits excavated to a depth of 30 cm at five positions (east, south, west, north, and center) within each plot (Huang et al., 2022). Soil samples were obtained from three depth layers (0–10 cm [upper layer], 10–20 cm [middle layer], and 20–30 cm [lower layer]), with five replicate samples taken for each layer and mixed into a single composite sample. Fresh soil samples were weighed, sealed, and transported to the laboratory. In the laboratory, the samples were air-dried and passed through a 100-mesh sieve before chemical analysis. The following soil chemical properties were measured: pH, soil organic carbon (SOC), total nitrogen (TN), total phosphorus (TP), total potassium (TK), available phosphorus (AP), nitrate nitrogen (NO^-^_3_-N), and available potassium (AK). The soil properties were measured using standard analytical methods: soil pH was measured using a pH meter; soil organic carbon was determined using the potassium dichromate oxidation-external heating method; total nitrogen was quantified using the Kjeldahl method; total phosphorus was measured using the molybdenum-antimony colorimetric method; total potassium was measured using flame photometry; and available phosphorus was extracted with NaHCO_3_ and determined by the molybdenum-antimony anti-colorimetric method; nitrate nitrogen was determined by ultraviolet spectrophotometry, while available potassium was extracted using ammonium acetate (NH_4_OAc) and measured using flame photometry.

### 2.4 Calculation of species importance values

In each quadrat, the number of individuals per species was counted, and relative abundance was calculated as (number of individuals of each species/total number of individuals of all species) × 100%. Across all quadrats, the occurrence frequency of each species was recorded, and relative frequency (RF) was calculated as (occurrence frequency of each species/the sum of the occurrence frequencies of all species) × 100%. Diameter at breast height (DBH) of tree individuals within each quadrat was measured, and basal area was calculated. Relative dominance was then determined as (basal area of a given tree species/total basal area of all tree species) × 100%. Vertical projected cover of each species was visually estimated, and relative cover was calculated as (cover of each species/total cover of all species) × 100%. The important value (IV) was calculated as follows:


Tree layer IV=(relative abundance+relative frequency+relative dominance)/3

Shrub layer IV=(relative abundance+relative frequency+relative cover)/3

### 2.5 Leaf sampling and measurement of functional traits

Dominant species were selected for leaf sampling based on the importance values (IV) of woody plants within the study region, yielding 49 selected woody species (tree IV > 0.5, shrub IV > 0.3). Following the protocols in the New Handbook for Standardised Measurement of Plant Functional Traits Worldwide (Pérez-Harguindeguy et al., 2013), target leaves were obtained from the study area during peak growth (July–August 2025). In each plot, 3–5 mature individuals were sampled for each target species, while all available individuals were sampled when fewer than three were present. For each individual, 3–5 third-order branches were selected from the south-facing part of the mid-canopy, with similar vigor and no visible disease, from which 15–30 intact mature leaves were collected. A total of 21 leaf functional traits were measured in this study (fresh leaf mass was mainly used to calculate derived traits), including leaf area (LA), specific leaf area (SLA), specific leaf weight (SLW), leaf water content (LWC), leaf thickness (LT), leaf dry weight (LDW), leaf dry matter content (LDMC), and leaf tissue density (LTD); Leaf photosynthetic pigment traits, including chlorophyll a (Chl a), chlorophyll a/b ratio (Chl a/Chl b), chlorophyll b (Chl b), and total chlorophyll content (Total Chl), were measured; leaf nutrient and stoichiometric traits, including leaf nitrogen content (LNC), leaf potassium content (LKC), leaf phosphorus content (LPC), leaf magnesium content (LMC), leaf carbon content (LCC), leaf iron content (LIC), leaf carbon-to-nitrogen ratio (C:N), leaf carbon-to-phosphorus ratio (C:P), and leaf nitrogen-to-phosphorus ratio (N:P).

Leaf thickness and fresh mass were measured in the field after the leaf surface was gently cleaned, and fresh mass was weighed using an electronic balance; values were then averaged. Avoiding the midrib, a digital thickness gauge was used to measure the leaf thickness at three points (proximal, middle, and distal) to obtain a mean value. Following petiole removal, leaf samples from each individual plant were separately bagged and transported to the laboratory for subsequent analyses. Leaf area was measured using a leaf area scanner. The samples were then oven-dried at 70 °C for 72 h until a constant mass was reached, after which dry mass was determined. After the dried samples were ground and sieved, carbon (C) and nitrogen (N) concentrations were measured using an automated elemental analyzer (vario MICRO cube, Elementar). The derived functional traits were calculated as follows: leaf dry matter content (dry mass/fresh mass), specific leaf area (leaf area/dry mass), specific leaf weight (dry mass/leaf area), leaf water content [(fresh mass − dry mass)/dry mass, %], and leaf tissue density (dry mass/volume).

### 2.6 Calculation of plant functional diversity

In this study, functional dispersion (FDis), functional richness (FRic), and Rao’s quadratic entropy (RaoQ) were used to quantify functional diversity, as follows: Functional dispersion (FDis) measures abundance-weighted dispersion of species in multidimensional trait space and reflects functional trait variability within a community (Laliberté and Legendre, 2010); Functional richness (FRic) quantifies the volume of niche space occupied by species within a community. Higher FRic values indicate broader use of available resources within the community; Rao’s quadratic entropy (RaoQ) is based on entropy theory and reflects the uniformity of trait values within a community (Rao, 1982), with higher values indicating weaker interspecific competition and lower niche overlap among species. The community-weighted mean (CWM) represents the abundance-weighted average of plant functional traits within a community and is calculated using species-specific trait values and their relative abundances (de Bello et al., 2005).

### 2.7 Driving factor data

A total of 23 drivers were selected in this study (**Table 1**), including soil properties, meteorological and environmental conditions, anthropogenic disturbances, and landscape pattern variables, as follows:

**Table 1:** Selection of driver variables.

Data type	Variables	Unit	Data sources
Soil Data	Soil pH (pH)	/	This study (laboratory measurements)
	Soil Organic Carbon (SOC)	g·kg^−1^	
	Total Nitrogen (TN)	g·kg^−1^	
	Total Phosphorus (TP) Total Potassium (TK) Available Phosphorus (AP) Nitrate Nitrogen (NO^-^_3_-N)	g·kg^−1^ g·kg^−1^ mg·kg^-1^ mg·kg^-1^	
	Available Potassium (AK)	mg·kg^-1^	
Meteorological and Environmental data	Elevation (ELE)	m	Data Platform for Resource and Environmental Sciences, Chinese Academy of Sciences(https://www.resdc.cn/)
	Mean Annual Temperature (MAT)	°C	National Earth System Science Data Center of the Chinese Academy of Sciences(https://www.geodata.cn/main/)
	Mean Annual Precinitation (MAP)	mm	
	Mean Ammual Relative Humidiy (MARH) Total Solar Radiation (TSR)	% kJ·m^−2^·d^−1^	
Anthropogenic Disturbance Data	People Density (PD)	people·km^−2^	Data Platform for Resource and Environmental Sciences, Chinese Academy of Sciences(https://www.resdc.cn/)
	Night Light Index (NLI)	/	
	Distance from Quadats to Main Water System (DMW)	m	
	Dictanee from Quadrats to Main Road (DMR)	m	
Landscape Data	Number of Patches (NP)	/	This study was carried out using existing land-use status data for calculation and analysis.
	Largest Patch Index (LPI)	%	
	Landscape Shape Index (LSI)	/	
	Edge Density (ED)	M·ha^−1^	
	Landscape Shannon’s Diversity Index (SHDI)	/	
	Aggregation Index (AI)	%

1. Soil data: After fresh soil samples were weighed, the samples were sealed and sent for chemical analysis, including soil pH (pH), soil organic carbon (SOC), total nitrogen (TN), total phosphorus (TP), total potassium (TK), available phosphorus (AP), nitrate nitrogen (NO^-^_3_-N), and available potassium (AK).
2. Meteorological and environmental data: We obtained the 2022–2024 elevation (ELE) for the study area from the Resource and Environment Science Data Platform, managed by the Chinese Academy of Sciences; Meteorological data for the study area from 2022 to 2024—including mean annual temperature (MAT), mean annual precipitation (MAP), mean annual relative humidity (MARH), and total solar radiation (TSR)—were obtained from the National Earth System Science Data Center, Chinese Academy of Sciences (https://www.geodata.cn/main/).
3. Anthropogenic disturbance data: We used population density (PD) and nighttime light index (NLI) data for the study area in 2024, which were obtained from the Resource and Environment Science Data Platform managed by the Chinese Academy of Sciences. The distance from the plot to the main water system (DMW) and the distance from the plot to the main road (DMR) were both obtained using the Euclidean distance analysis tool in ArcGIS 10.8 software.
4. Landscape data: To describe the land-use conditions and landscape features of the study area, six landscape pattern metrics were selected across three core dimensions—patch scale, shape regularity, and spatial distribution—including patch number (NP), largest patch index (LPI), landscape shape index (LSI), edge density (ED), landscape Shannon diversity index (SHDI), and aggregation index (AI).

### 2.8 Data processing

Data for both plant leaf functional traits and drivers were managed in Microsoft Excel 2019. Normality was tested using the Shapiro–Wilk test in SPSS. Because the data violated parametric assumptions, a nonparametric Wilcoxon test was used to test differences between groups. Spearman’s rank correlation was used to evaluate the pairwise relationships between environmental variables and functional diversity indices, including RaoQ, FDis, and FRic. Drivers with strong collinearity were removed according to the variance inflation factor (VIF > 10), and RDA was then used to evaluate how much the driving factors explained functional diversity. Principal component analysis (PCA) was performed on soil factors to extract composite variables representing soil fertility. Structural equation modeling (SEM) was applied to establish a model in which functional diversity indices were treated as endogenous variables, while the key drivers derived from RDA were treated as exogenous variables. Variables that exhibited significant or marginally significant associations with functional diversity in the RDA (*p* < 0.10) were retained as candidate exogenous variables for the SEM. The effects and pathways through which key drivers influence functional diversity were evaluated using goodness-of-fit, including the chi-square/degrees of freedom (χ^2^/df), root mean square error of approximation (RMSEA), comparative fit index (CFI), standardized root mean square residual (SRMR), and the variance in diversity accounted for by exogenous variables (R^2^). Statistical calculations and visualizations were performed using R (version 4.5.2), Canoco 5.0, SPSS, and OriginPro 2026.

## 3 Results

### 3.1 Functional diversity characteristics of plant communities across volcanic lava landscapes in northern Hainan

#### 3.1.1 Functional traits of woody plants

**Figure 2:**
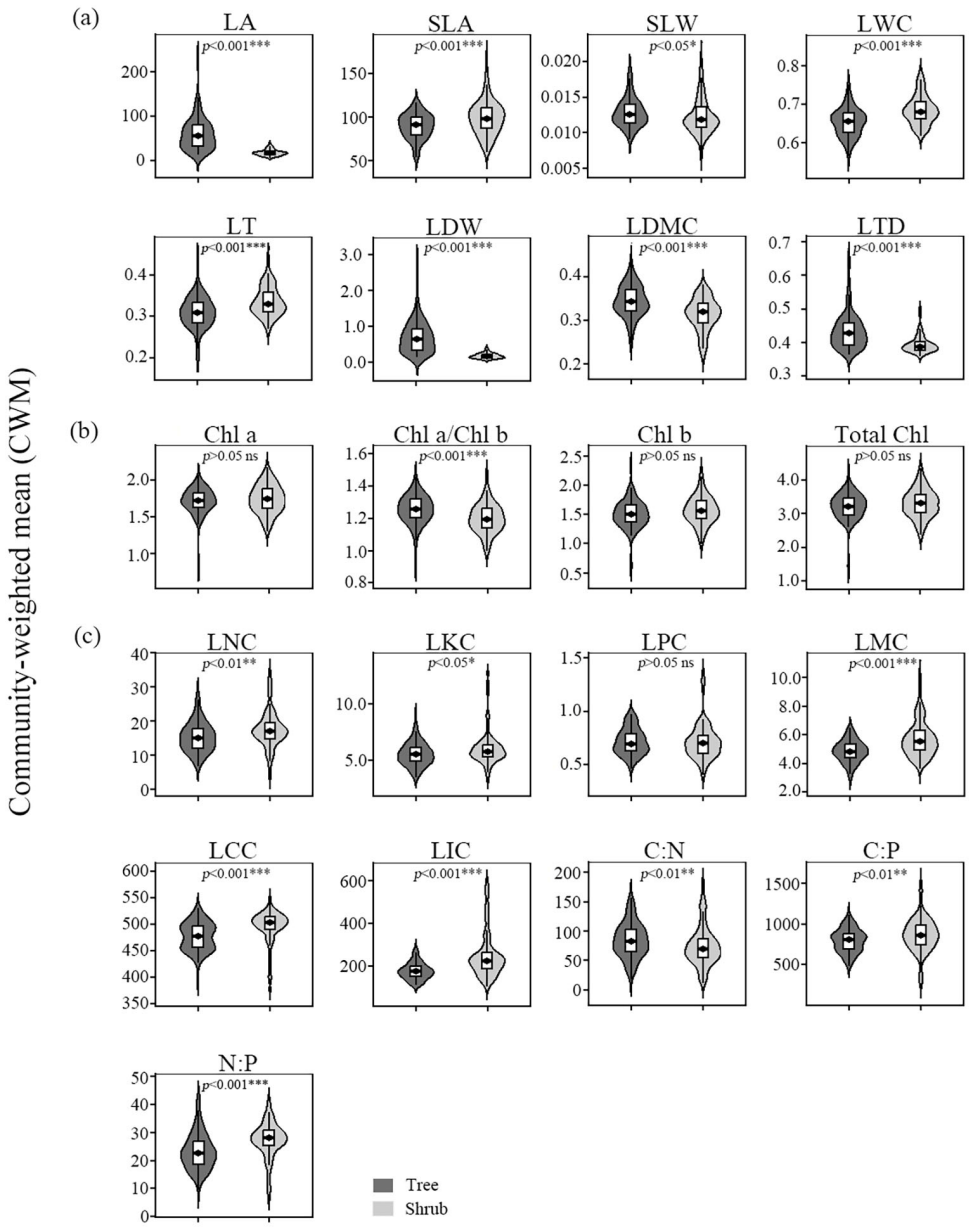
Analysis of differences in community-weighted means among different growth forms of woody plants. *Significant difference at (*p* < 0.05); **highly significant difference at (*p* < 0.01); ***very highly significant difference at (*p* < 0.001); ns, not significant. The same applies below. Panels **(a–c)** represent leaf physical structural traits, leaf photosynthetic pigment traits, and leaf nutrient and stoichiometric traits, respectively.

**Figure 2** shows that the CWM values of functional traits differed among growth forms of woody plants in the study area. Wilcoxon tests showed that trees exhibited significantly higher CWM values for LA, LDW, LDMC, LTD, and Chl a/Chl b than shrubs (*p* < 0.001). By contrast, the CWM values for the tree layer were significantly lower than those observed for the shrub layer for several key functional traits, including SLA, LWC, LT, LMC, N:P, LCC, and LIC (*p* < 0.001); similarly, LNC, C:N, and C:P were significantly lower than those of the shrub layer (*p*< 0.01), whereas the CWMs of SLW and LKC were also lower (*p* < 0.05). No significant differences were observed between trees and shrubs in the CWMs of LPC, Chl a, Chl b, and Total Chl (*p* > 0.05).

#### 3.1.2 Functional diversity patterns of woody plant communities

**Figure 3:**
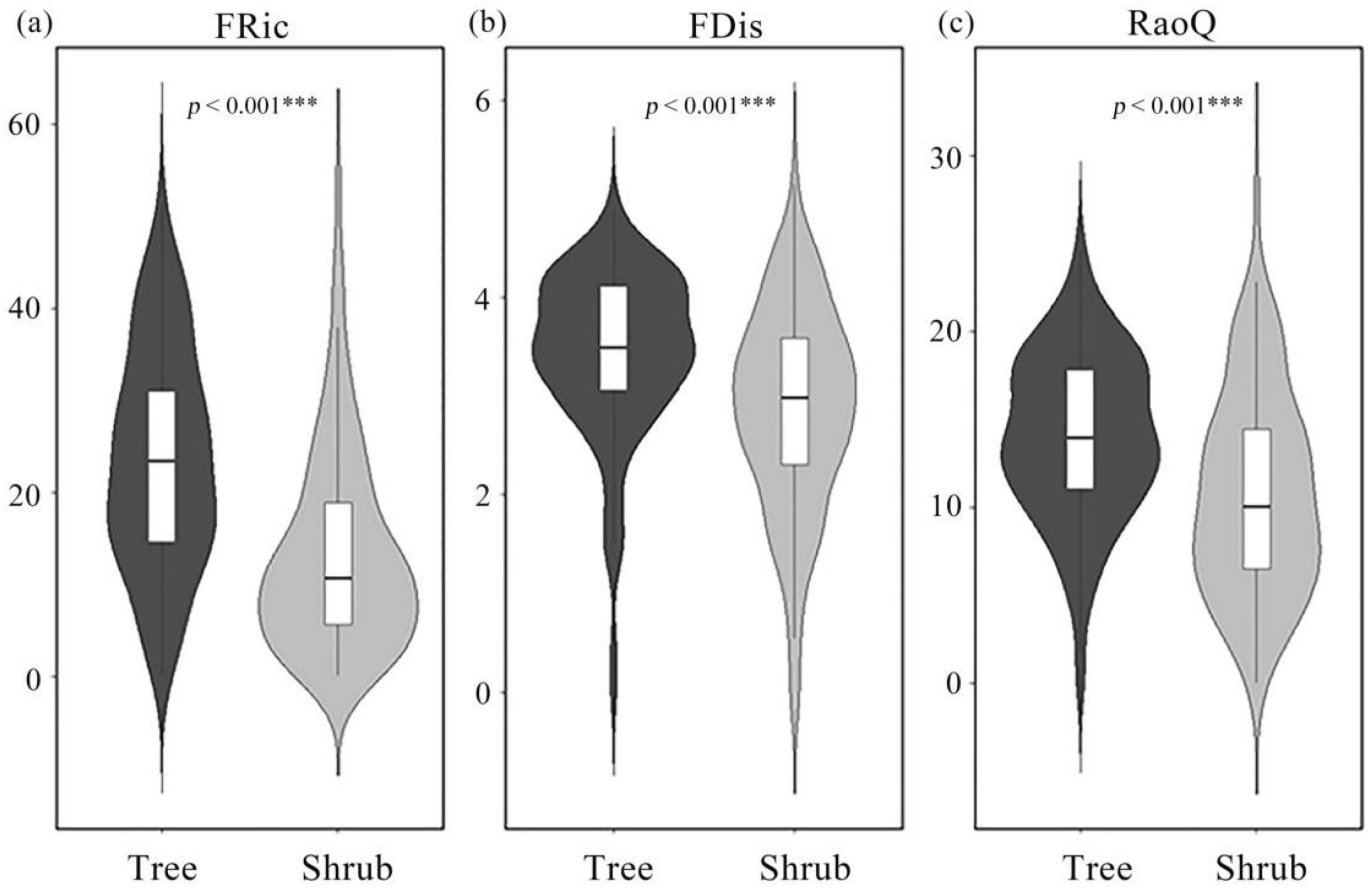
Differences in functional diversity indices among woody plant growth forms. Panels **(a–c)** represent functional richness, functional dispersion, and Rao’s quadratic entropy, respectively. *** Extremely significant difference at (p<0.001).

As shown in **Figure 3**, the use of non-parametric Wilcoxon rank-sum tests showed highly significant differences between the tree and shrub layers in FRic, FDis, and RaoQ (*p* < 0.001). In addition, the FRic, FDis, and RaoQ of trees were all significantly higher than those of shrubs (*p* < 0.001).

### 3.2 Relationships between plant community functional diversity and driving factors

#### 3.2.1 Correlation between functional diversity and driving factors

**Figure 4:**
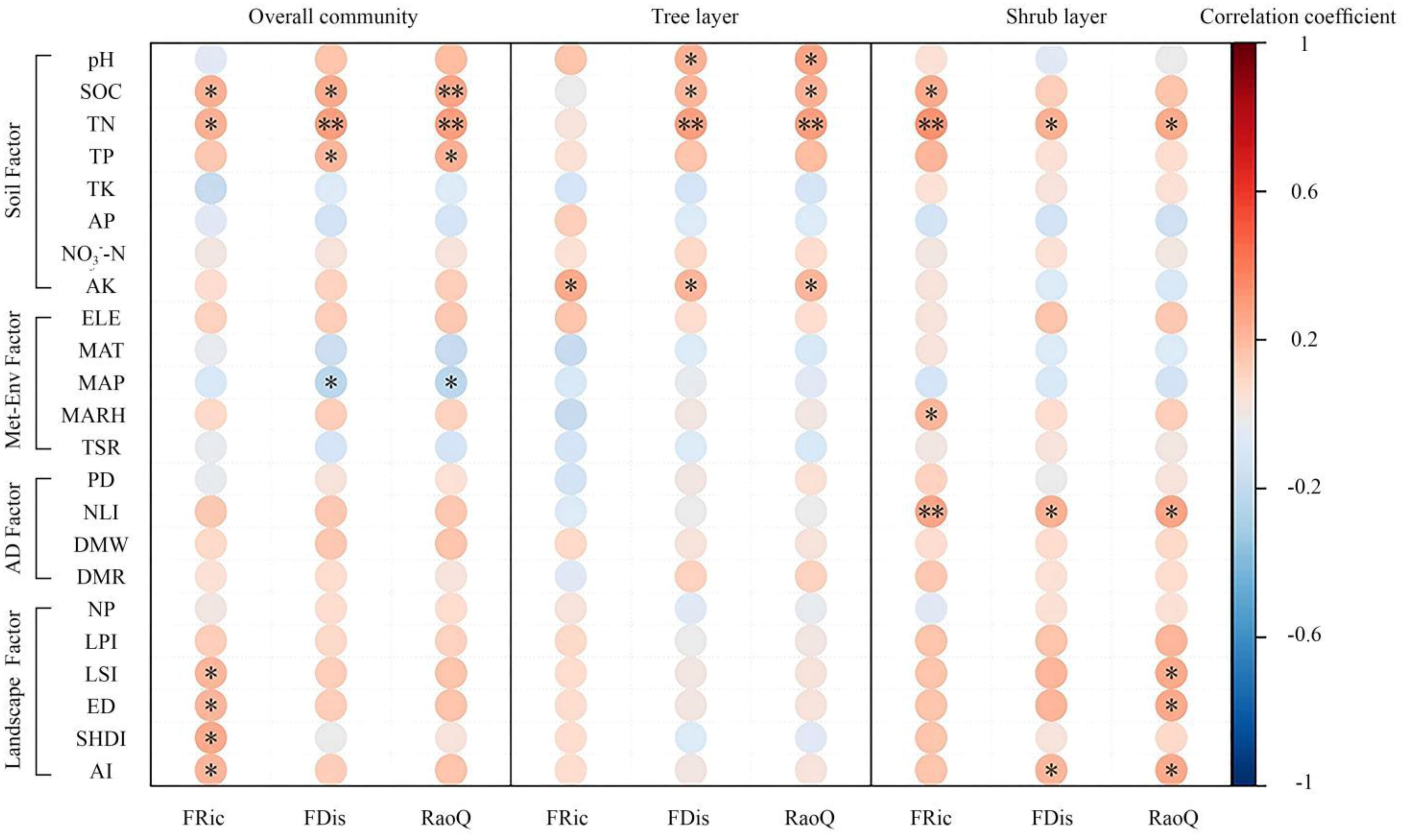
Spearman correlation analysis between plant community functional diversity and its driving factors. *indicates a significant correlation (*p* < 0.05), **indicates a highly significant correlation (*p* < 0.01).

As shown in the Spearman correlation matrix (**Figure 4**), FRic for the whole community was significantly and positively correlated with SOC, TN, LSI, ED, SHDI, and AI (*p* < 0.05). FDis was strongly and positively correlated with TN (*p* < 0.01) and positively correlated with SOC and TP (*p* < 0.05). RaoQ was strongly and positively correlated with SOC and TN (*p* < 0.01) and positively correlated with TP (*p* < 0.05), whereas both FDis and RaoQ were negatively correlated with MAP (*p* < 0.05).

At the level of vertical community strata, tree-layer FRic was positively correlated only with AK (*p* < 0.05). Tree-layer FDis and RaoQ were strongly and positively correlated with TN (*p* < 0.01) and positively correlated with pH, SOC, and AK (*p* < 0.05). In the shrub layer, FRic was strongly and positively correlated with TN and NLI (*p* < 0.01) and positively correlated with SOC and MARH (*p* < 0.05). Shrub-layer FDis and RaoQ were both positively correlated with TN, NLI, and AI (*p* < 0.05), and RaoQ was also positively correlated with LSI and ED (*p* < 0.05).

#### 3.2.2 Redundancy analysis of functional diversity and driving factors

**Figure 5:**
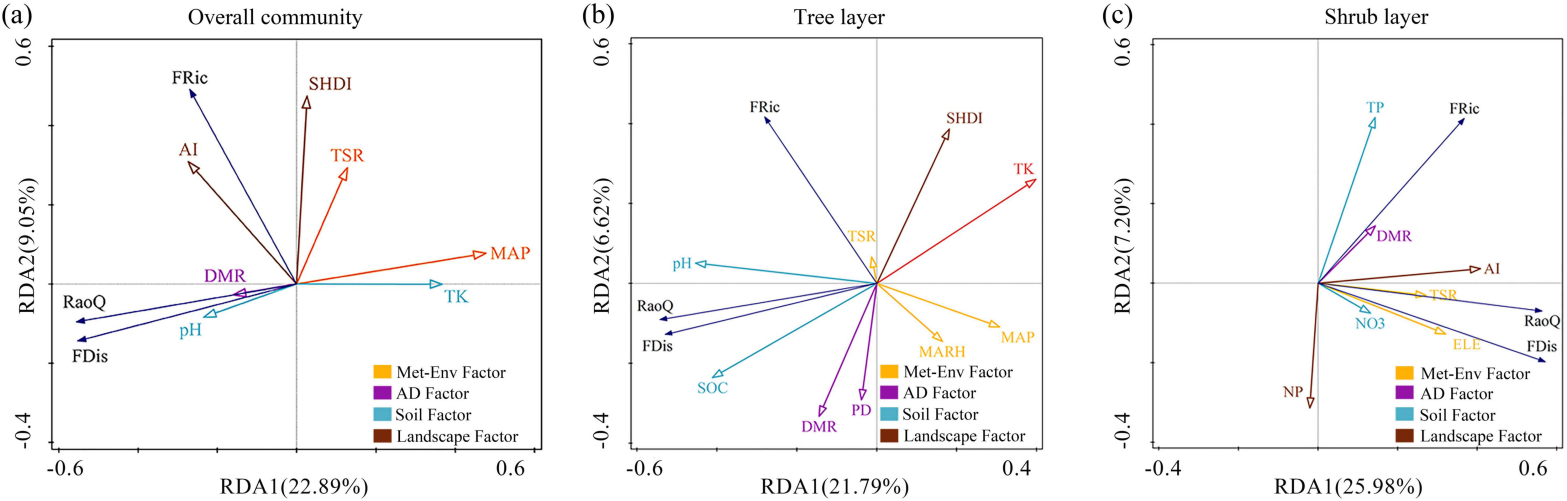
Redundancy analysis of plant community functional diversity and driving factors. Panels **(a–c)** show RDA results for the whole community, tree layer, and shrub layer, respectively. The image displays the main driving factors retained in the analysis.

For the overall community (**Figure 5a**), RDA axes 1 and 2 together explained 31.94% of the variation in functional diversity. Among the retained drivers, MAP had the largest relative contribution, explaining 5.3% of the variation (*p* < 0.05), followed by SHDI, TK, and DMR, which explained 5.0%, 3.9%, and 3.1%, respectively. The remaining drivers showed comparatively limited explanatory power.

For the tree layer (**Figure 5b**), the first two RDA axes explained 28.41% of the variation. Soil pH made the strongest contribution to tree-layer functional diversity, explaining 4.5% of the variation (*p* < 0.05), followed by TK, which explained 4.3% (*p* < 0.05). Other drivers contributed relatively little to the explained variation.

For the shrub layer (**Figure 5c**), the first two RDA axes accounted for 33.18% of the variation. AI was the strongest driver of shrub-layer functional diversity, explaining 4.3% of the variation (*p* < 0.05), followed by DMR and NP, which explained 4.1% and 3.8%, respectively (both *p* < 0.05). The explanatory power of the remaining variables was comparatively weak.

### 3.3 Path effects of key driving factors on plant functional diversity

**Figure 6:**
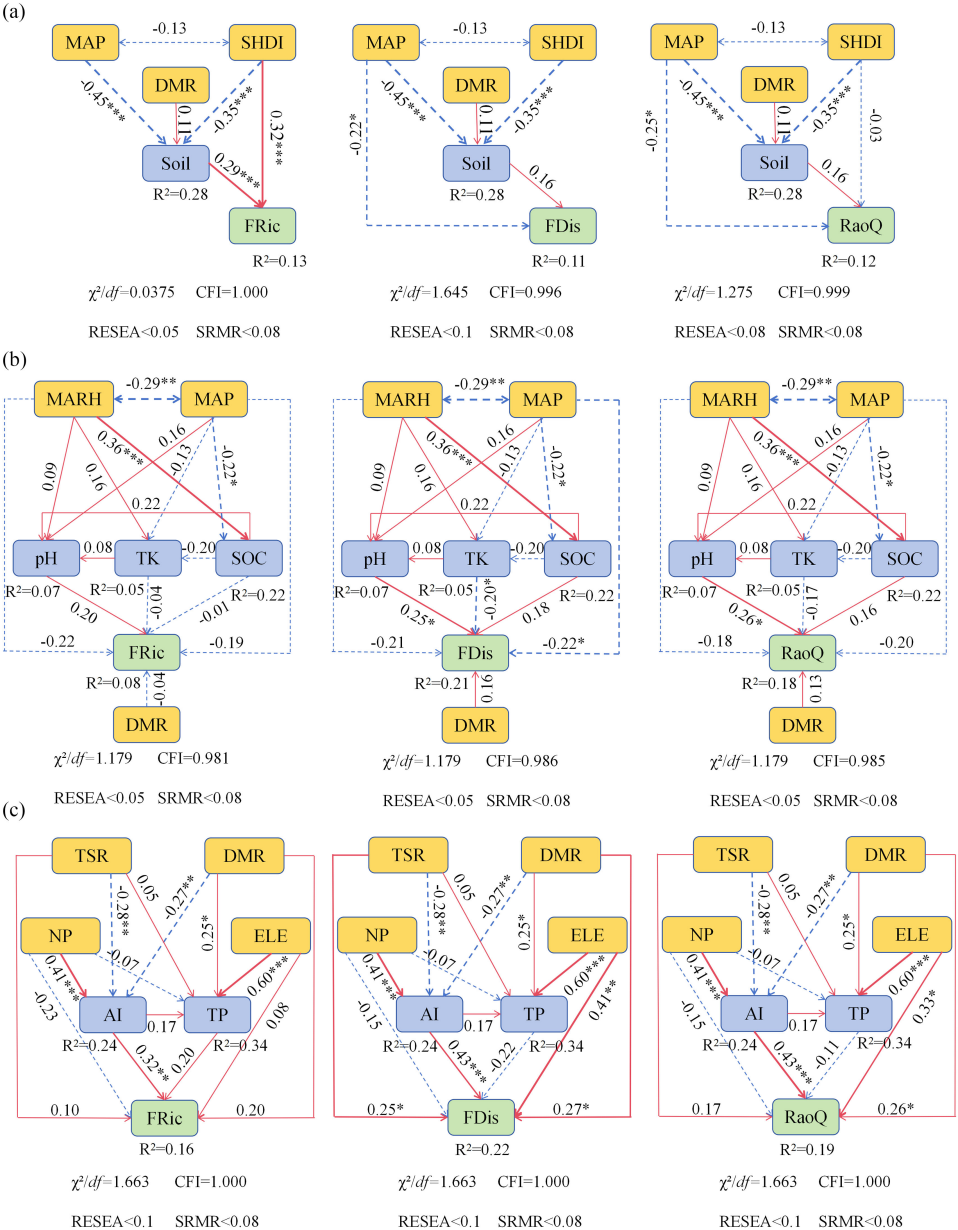
Pathways through which key driving factors influence community functional diversity. Solid lines indicate significant relationships at *p* < 0.01, and dashed lines indicate significant relationships at *p* < 0.05. Panels **(a–c)** represent the whole community, tree layer, and shrub layer, respectively. χ^2^ denotes the chi-squared statistic for goodness of fit; df denotes degrees of freedom; χ^2^/df denotes the ratio of chi-squared to degrees of freedom; RMSEA denotes the root mean square error of approximation; and CFI denotes the comparative fit index.

For pathways affecting whole-community functional diversity (**Figure 6a**), SHDI had a significant positive association with FRic and represented the main pathway (β = 0.318, *p* < 0.01), followed by soil factors (Soil) (β = 0.286, *p* < 0.01). MAP was negatively associated with Soil and showed the strongest pathway related to soil variation (β = −0.454, *p* < 0.001). SHDI was also negatively associated with Soil (β = −0.351, *p* < 0.001), whereas DMR showed a weak and non-significant association with Soil (β = 0.109, *p* > 0.05). MAP was negatively associated with both FDis and RaoQ (β = −0.224, *p* < 0.05; β = −0.251, *p* < 0.05), whereas Soil and SHDI showed no significant associations with FDis or RaoQ (all *p* > 0.05).

For tree-layer functional diversity (**Figure 6b**), MARH was positively associated with SOC (β = 0.363, *p* < 0.001). Through its association with higher SOC, MARH was indirectly linked to lower FRic and higher FDis and RaoQ. By contrast, MAP was negatively associated with SOC (β = −0.217, *p* < 0.05), suggesting an indirect pathway through reduced SOC that was related to lower FRic but higher FDis and RaoQ.

For shrub-layer functional diversity (**Figure 6c**), ELE and DMR were positively associated with TP (β = 0.605, *p* < 0.001; β = 0.255, *p* < 0.05). AI was also positively associated with FDis, FRic, and RaoQ. Through higher TP levels, these drivers were indirectly linked to higher FRic and RaoQ but lower FDis (β = 0.425, *p* < 0.001; β = 0.317, *p* < 0.01; β = 0.429, *p* < 0.001).

## 4 Discussion

### 4.1 Functional diversity patterns of woody plant communities in the northern Hainan volcanic lava region

Functional richness (FRic) quantifies the extent of niche or habitat space occupied by species. Higher FRic values generally indicate broader resource use and may be associated with higher productivity and ecosystem stability (Petchey and Gaston, 2002). In this study, shrub-layer FRic was significantly lower than tree-layer FRic, suggesting that tree communities occupy broader niche space and use a wider range of ecological strategies, whereas shrub-layer functional traits are more convergent. This pattern is consistent with observations from tropical rainforest communities in Xishuangbanna (Chen et al., 2024) and reflects growth-form differences in functional diversity. Higher niche differentiation and more diverse resource-use strategies in tree communities may also explain why tree-layer FDis was significantly higher than shrub-layer FDis. Tree species vary more widely in plant size, resource-use strategy, and life-history traits, allowing them to occupy a broader functional space (Díaz et al., 2016). In contrast, shrubs persist beneath the tree canopy, where light limitation and resource competition may constrain trait variation. Thus, the lower shrub-layer FDis may be related to stronger understory filtering, whereas long-term exposure of canopy trees to high light and variable nutrient availability may promote differentiation along fast–slow growth strategies. The global plant economics spectrum provides a useful explanation for this pattern. Trees may occupy a wider range of positions along the trait spectrum, whereas understory shrubs tend to converge toward shade-tolerant and resource-acquisitive strategies under canopy shading, resulting in narrower trait variation and lower FDis (Reich, 2014).

Across the volcanic lava landscapes of northern Hainan, co-occurring woody species with different growth forms showed clear differences in leaf functional traits. Tree CWM for SLA was significantly lower than shrub CWM, probably because canopy trees are exposed to stronger and more persistent light, where lower SLA may favor adaptation to high-light environments. In shaded understory habitats with lower light availability and higher humidity, shrubs with higher SLA may improve light capture and photosynthetic efficiency. These differences suggest that contrasting growth forms use different ecological strategies to adapt to the hot and humid conditions of the northern Hainan volcanic lava region (Chen et al., 2024). Tree CWM for LT was significantly lower than shrub CWM, consistent with previous evidence that dominant species differ in functional traits among plant life forms in the Kanas landscape (Wang et al., 2024). In shrubs, thicker leaves may help plants cope with understory environmental stress and contribute to soil and water conservation, thereby improving resistance to harsher conditions (Zhou et al., 2019). The chlorophyll a/b ratio was also significantly higher in trees than in shrubs. Wang et al. (2019) reported that the chlorophyll a/b ratio decreased under shaded conditions but increased in high-light environments, indicating physiological acclimation to light variation. In the northern Hainan volcanic lava region, the canopy and understory differ substantially in light conditions. Trees exposed to high light may benefit from a higher chlorophyll a/b ratio, which improves light-use efficiency and reduces photodamage, whereas shrubs in low-light habitats may rely on a lower chlorophyll a/b ratio to enhance light harvesting, consistent with the adaptive traits of tropical understory plants (Sun et al., 2022).

### 4.2 Relationships between plant community functional diversity and environmental drivers

Soil resources are important drivers of plant growth and community development. They provide the basic substrate for plant survival and regulate key biogeochemical processes, including nutrient cycling and water retention (Valencia et al., 2022). Our results show that multidimensional functional diversity indices were positively associated with SOC, TN, and TP. This pattern may occur because sufficient nitrogen promotes leaf cell division and elongation, increases leaf area and SLA, and enhances photosynthetic capacity, thereby supporting plant growth (Siefert and Ritchie, 2016). Higher phosphorus availability may also increase resource allocation to aboveground growth and accelerate plant height growth (Xiao et al., 2020). Tree-layer FRic was positively associated only with AK, whereas shrub-layer FRic was positively associated with SOC and TN, indicating growth-form differences in response to environmental gradients. These differences may reflect contrasting resource-acquisition strategies. The shrub layer is usually more strongly influenced by surface soil nutrients and moisture, and resource heterogeneity may promote niche differentiation (Villéger et al., 2008). Therefore, the shrub layer may be more sensitive to variation in surface resources, although the underlying assembly processes require further testing. TP and TN also affected FDis and RaoQ, consistent with findings for shrub-layer functional diversity in Rhododendron latoucheae communities in the Jinggang Mountains (Xiang et al., 2019). In that region, favorable hydrothermal conditions and relatively abundant nutrients allow variation in soil nitrogen and phosphorus to influence RaoQ and FDis by altering leaf trait differentiation. By contrast, the northern Hainan volcanic lava region is constrained by volcanic parent material and shallow soils with limited water- and nutrient-retention capacity. In addition, Spasojevic and Suding (2012) suggested that functional diversity patterns are unlikely to be explained by a single community assembly mechanism, because multiple ecological processes may operate simultaneously along different trait axes.

Plant trait variation is closely related to environmental heterogeneity, which supports species coexistence in changing environments (Ameztegui et al., 2017). Previous studies indicate that community functional structure is usually shaped by abiotic filtering, biotic interactions, and spatially structured assembly processes rather than by a single dominant environmental driver (Kraft et al., 2015; Wiegand et al., 2017; Shinohara et al., 2023). Water availability has been identified as an important driver of plant species richness (Koerner and Collins, 2014) and may therefore influence community functional diversity. Consistent with Zuo et al. (2021), who found that different facets of functional diversity respond differently to moisture gradients in arid shrublands and grasslands, our results suggest that MAP is associated with functional diversity across the studied communities. Lower precipitation may restrict photosynthesis and energy utilization, thereby reducing functional diversity. Precipitation may also regulate physiological processes and water-use strategies, which can modify species niche differentiation and influence community functional diversity (Wang et al., 2022). In this study, SHDI was significantly related to functional diversity and made a relatively large contribution to community functional diversity, although the overall explanatory power of individual drivers was limited. Greater landscape heterogeneity may enhance habitat diversity, resource availability, and niche space, thereby promoting the coexistence of species with different functional traits and expanding community functional trait space (Villéger et al., 2008).

### 4.3 Path effects of key driving factors on plant community functional diversity

Soil factors were positively associated with plant functional diversity and may regulate trait structure both directly and indirectly as part of broader community assembly processes. Soil physicochemical properties determine local resource availability and can favor different ecological strategies under different soil environments (Soong et al., 2020). This finding is consistent with Lavorel and Garnier (2002), who showed that soil resources influence community functional structure by shaping combinations of plant functional traits. Soil conditions may affect functional diversity through two main pathways. First, soil nutrient conditions can directly influence functional diversity responses. Under water-limited conditions, plants often show higher leaf tissue density and lower SLA and plant height, which can promote trait convergence and reduce FRic (Zhou et al., 2023). Second, functional diversity also responds to soil carbon, nitrogen, and phosphorus contents. Nutrient limitation may restrict species coexistence, whereas nutrient enrichment can increase SLA and leaf nitrogen and phosphorus concentrations (Li et al., 2016). Key drivers and their direct and indirect associations with functional diversity also differ between the tree and shrub layers. Because canopy trees are taller and have deeper root systems, they may buffer short-term environmental fluctuations (Petchey and Gaston, 2006). By contrast, the shrub layer has shallower roots and faster turnover, making it more sensitive to changes in topsoil resources. Environmental factors may therefore influence shrub-layer functional diversity indirectly through changes in resource availability and interspecific interactions (Villéger et al., 2008).

MAP was negatively correlated with FDis and RaoQ and showed an indirect association with functional diversity through soil factors. Variation in precipitation regimes may influence community taxonomic composition and multidimensional functional structure by regulating soil resource availability and spatial environmental heterogeneity (Vandewalle et al., 2010). As an important climatic factor, precipitation may affect community functional structure directly and indirectly through changes in soil conditions, forming a potential climate–soil–functional diversity pathway. Some functional traits and multidimensional functional diversity indices showed monotonic decreases or non-significant changes along the increasing precipitation gradient, suggesting non-linear or negative sensitivity to local moisture availability (Liu et al., 2025). Anthropogenic disturbance did not show uniformly significant effects on functional diversity, possibly because communities can maintain functional structural stability under certain ecosystem conditions and disturbance intensities. This result is consistent with previous findings that disturbance may alter species ecological groups without necessarily producing large changes in functional diversity, as community-level functional replacement can maintain functional homeostasis (Lü et al., 2009). Overall, under low-to-moderate disturbance, plant communities may maintain functional diversity through compensatory functional replacement, whereas high-intensity disturbance may exceed these internal buffers and alter community functional structure. SEM helped clarify the pathway network through which environmental drivers are associated with woody plant functional diversity in the northern Hainan volcanic lava region. Different drivers influenced functional diversity through both direct and indirect pathways. For example, SHDI was positively associated with FRic and may indirectly enhance overall functional diversity through its relationship with soil factors. This result is consistent with studies showing that landscape heterogeneity can promote biodiversity (Stein et al., 2014). Greater landscape diversity may increase habitat diversity and resource heterogeneity, expand niche space, and support the coexistence of functionally divergent species, thereby increasing functional richness (Villéger et al., 2008).

## 5 Conclusion

In this study, correlation analysis, RDA, and SEM were used to identify key drivers of plant functional diversity in the northern Hainan volcanic lava region and to assess their associations with functional diversity. The main conclusions are as follows:

1. Clear differences in CWM functional traits and functional diversity among woody plant growth forms indicate that plants with contrasting growth forms may adopt different ecological strategies in response to environmental conditions. These differences may enhance competitive performance and allow species to occupy broader ecological niches.
2. In the northern Hainan volcanic lava region, plant community functional diversity was jointly associated with soil properties, meteorological and environmental conditions, anthropogenic disturbance, and landscape factors. Soil variables were related to functional diversity through both direct and indirect pathways. These drivers may regulate plant growth, competitive interactions, niche partitioning, and environmental adaptability, thereby influencing the functional structure of plant communities to varying degrees.

Although this study provides new evidence on woody plant functional diversity in the northern Hainan volcanic lava region, several limitations remain. Key functional dimensions, including stem structural traits, hydraulic strategies, belowground nutrient uptake, reproductive traits, and seed dispersal mechanisms, were not included, which limits the ability to fully characterize the multidimensional functional niches of woody plant communities. Because this study was based on observational data, the inferred mechanisms should be further tested in future work. In addition, potentially important factors such as soil microbial communities, plant–soil feedbacks, and interspecific interactions were beyond the scope of this study; their omission may have contributed to the unexplained variation in functional diversity patterns. Although the sampling design was intended to reduce spatial dependence through a plot layout designed to avoid clustering, the possible influence of latent spatial structure on model inference, coefficient estimation, and significance testing cannot be completely excluded. Future research should incorporate formal spatial autocorrelation diagnostics, spatially explicit modeling approaches, and sensitivity analyses to quantify, control for, and transparently report spatial dependence; it should also combine long-term monitoring with multi-organ functional trait measurements to develop a more comprehensive understanding of the mechanisms shaping functional diversity in woody plant communities in the volcanic lava landscapes of northern Hainan.

## Data Availability

The raw data supporting the conclusions of this article will be made available by the authors, without undue reservation.
