# Supplementary material for: Functional diversity of woody plant communities and its driving factors in the northern Hainan volcanic lava region
Source: Front Plant Sci. 2026 Jul 29;17:1888449. doi: 10.3389/fpls.2026.1888449 (PMC13462395; doi:10.3389/fpls.2026.1888449)
Supplement: Supplementary file 1 [file Table1.pdf]

1 Supplementary Material

1 Selection of Dominant Woody Species of Different Growth Forms in the Study Area

Tree			Shrub		
Species	Scientific Latin name	IV	Species	Scientific Latin name	IV
Litchi	Litchi chinensis	13.55	Boxleaf atalantia	Atalantia buxifolia	4.02
Longan	Dimocarpus longan	5.42	Red psychotria	Psychotria asiatica	3.40
Chinaberry tree	Aphanamixis polystachya	4.98	Chinese desmos	Desmos chinensis	2.24
Upas tree	Antiaris toxicaria	3.80	Dwarf ardisia	Ardisia humilis	2.21
Kandelia tree	Lannea coromandelica	3.63	Smooth-fruited ventilago	Malaisia scandens	2.12
Microcos	Microcos paniculata	3.19	Pearl maesa	Micromelum falcatum	2.07
Opposite-leaved fig	Ficus hispida	2.99	Orange jasmine	Clausena excavata	1.82
Jackfruit	Artocarpus heterophyllus	2.74	Lau's polyalthia	Polyalthia suberosa	1.59
Chinese pistache	Streblus asper	2.60	Wallich's tarennoidea	Tarenna attenuata	1.58
Ardisia tree	Alangium salviifolium	2.36	Cochinchina blastus	Blachia pentzii	1.45
Chukrasia	Chukrasia tabularis	2.14	Orangeberry	Glycosmis pentaphylla	1.38

Chinaberry	Melia azedarach	1.94	Cobbe allophylus	Allophylus viridis	1.33
Silver wood	Aporosa dioica (Roxb.)	1.92	Rugose alchornea	Alchornea rugosa	1.31
Common ardisia	Wrightia pubescens	1.67	Cochinchina phyllanthus	Taxotrophis taxoides	1.29
Litsea species	Litsea glutinosa (Lour.)	1.42	Hainan ixora	Ixora hainanensis	1.22
Mallotus	Mallotus philippensis	1.36	Avicenna prickly ash	Zanthoxylum laetum	0.94
Wampee	Clausena lansium	1.36	Hairy persimmon	Diospyros strigosa	0.90
Trema	Kleinhovia hospita	1.16	Procumbent nightshade	Solanum procumbens	0.85
Wild pomegranate	Catunaregam spinosa	1.15	Java brucea	Brucea javanica	0.82
Water Syzygium	Syzygium nervosum	1.10	Shrubby breynia	Breynia fruticosa	0.8
Cratoxylum	Cratoxylum cochinchinense	0.97	Cyrtophyllum clerodendrum	Clerodendrum cyrtophyllum	0.78
Cat-tail tree	Markhamia stipulata	0.80	Shrubby oreocnide	Oreocnide frutescens	0.57
Smooth peach tree	Trevia nudiflora	0.72	Taiwan fig	Ficus formosana	0.51
Cycad fern	Aglaia lawii	0.55	Silver-leaved croton	Croton cascarilloides	0.49
			White-flowered ixora	Ixora henryi	0.32

2 Leaf functional traits and their ecological significance

Leaf functional trait	English abbreviation	Ecological interpretation	Unit	measurement reference
Leaf area	LA	Photosynthetic potential	cm^2	
Specific leaf area	SLA	Growth rate and resource-use efficiency	cm^2/g	
Specific leaf weight	SLW	Reflects structural investment and leaf longevity	g/cm^2	
Leaf water content	LWC	Water-use strategy and drought tolerance	%	Pérez-Harguindeguy et al., 2013
Leaf thickness	LT	Drought tolerance and light adaptation	mm	
Leaf dty weight	LDW	Leaf construction investment	g	
Leaf dry-matter content	LDMC	Drought tolerance and stress tolerance	g/g	
Leaf tissue density	LTD	Reflects mechanical strength and defense investment	g/cm^3	
Leaf chlorophyll a concentration	Chl a	The primary photosynthetic pigment, determining the plant's capacity for light energy capture.	g/kg	
The ratio of chlorophyll a to b	Chl a/Chl b	Reflects the degree of light stress and nitrogen limitation experienced by plants.	/	Lichtenthaler, 1987
Leaf chlorophyll b concentration	Chl b	An accessory photosynthetic pigment that enhances the utilization of low-light conditions.	g/kg	

Total chlorophyll concentration	Total Chl	Reflects the efficiency of photosynthetic processes.	g/kg	
Leaf nitrogen content	LNC	Reflects nutrient-use efficiency and plant growth potential.	g/kg	
Leaf potassium content	LKC	Plays a key role in controlling stomatal dynamics and maintaining cellular osmotic balance.	g/kg	
Leaf phosphorus content	LPC	Indicates the capacity for energy transfer and the rate of metabolic processes.	g/kg	
Leaf magnesium concentration	LMC	The central element of chlorophyll molecules, influencing photosynthetic efficiency.	g/kg	
Leaf carbon content	LCC	Indicates carbon sequestration capacity and biomass accumulation.	g/kg	Bao, 2000
Leaf iron concentration	LIC	Participates in the electron transport chain and chlorophyll biosynthesis, serving as an important micronutrient in photosynthesis.	mg/kg	
Leaf carbon to nitrogen ratio	C:N	Indicates the efficiency of nitrogen utilization	/	
Leaf carbon to phosphorus ratio	C:P	Indicates the efficiency of phosphorus utilization.	/	
Leaf nitrogen to phosphorus ratio	N:P	A key metric for determining nutrient limitation.	/	

3 Reference

Bao, S.D. (2000). Soil and Agricultural Chemistry Analysis.China Agriculture Press, Beijing.
Lichtenthaler, H. K. (1987). Chlorophylls and carotenoids: Pigments of photosynthetic biomembranes. *Methods in Enzymology.* 148, 350–382. doi: 10.1016/0076-6879(87)48036-1

Pérez-Harguindeguy, N., Díaz, S., Garnier, E., Lavorel, S., Poorter, H., Jaureguiberry, P.,et al. (2013). New handbook for standardised measurement of plant functional traits worldwide. *Australian Journal of Botany*. 61, 167-234. doi:10.1071/BT12225
